# Simplicial networks: a powerful tool for characterizing higher-order interactions

**DOI:** 10.1093/nsr/nwac038

**Published:** 2022-03-04

**Authors:** Dinghua Shi, Guanrong Chen

**Affiliations:** Department of Mathematics, College of Science, Shanghai University, China; Department of Electronic Engineering, City University of Hong Kong, China

In recent network research, more and more efforts have been devoted to studying higher-order interactions in complex networks that explain some intrinsic properties, discover hidden features that conventional mathematical tools cannot help to find and enable a clearer view of the real world. This article presents a perspective on the state-of-the-art progress in the field.

Edges in networks describe pairwise interactions between nodes, whereas for networks with higher-order interactions, hyperedges are used [1]. In many real-world networks, such as ecosystem networks, social networks and brain neural networks, higher-order interactions are ubiquitous [2]. To model various higher-order interactions, besides hypernetworks, there is a possibility of using the higher-order structure of the network itself, where they all depend on higher-order cycles. The shortest cycle is the triangle, which is largely involved in small-world networks. However, higher-order cycles have not been systematically investigated. In the study of network synchronizability, we introduced the concept of minimal cycle [3]. We then considered complete subgraphs [4] of different orders: node (0th order), edge (first order), triangle (second order), tetrahedron (third order) and so on, called cliques in graph theory or simplexes in algebraic topology, which are powerful tools for extending ordinary networks to simplicial networks with higher-order structures and interactions.

The main difference between a simplicial network and a hypernetwork is that, in a hyperedge there may not exist pairwise node interconnections, whereas all sub-simplexes are contained in the simplicial networks. Moreover, there are elegant and powerful mathematical theories and tools for computing and analysing simplicial networks. For a given simplicial network, the highest order of its simplexes is defined as the order of the network. For instance, one C. elegans neural network is a seventh-order simplicial network [5].

Various matrices in the simplicial networks provide a handy mathematical tool for studying higher-order interactions in networks. Specifically, for a network, suppose that the number of $d$-cliques (simplexes) is ${m_d}$. Let ${S_{cd}}$ ($c < d$) be the incidence matrix between a *c*-clique and a *d*-clique, in which an element is 1 if the *c*-clique belongs to the *d*-clique or 0 otherwise. In the matrix ${S_{0d}}S_{0d}^T$, the diagonal element ${s_{ii}}$ is the number of *d*-cliques containing node *i* and the non-diagonal elements together constitute the higher-order adjacency matrix ${A^{( {0, d} )}}$, which gives the higher-order transformation probability ${P^{( {0, d} )}}$ after normalization. Adding diagonal elements to make the matrix $ - {A^{( {0, d} )}}$ have zero row sums will result in a generalized Laplacian matrix, denoted ${L^{( {0, d} )}}$. The eigenvalues of matrix ${L^{( {0, d} )}}$ are all non-negative real numbers, satisfying $0= \lambda _1^{( {0, d} )}\!<\! \lambda _2^{( {0, d} )}\! \le\! \cdots \le \lambda _{{m_0}}^{( {0, d} )}$. Note that the *d*th-order simplicial network has $( {d + 1} )d/2$ incidence matrices and $( {d + 1} )d$ generalized Laplacian matrices, e.g. from $S_{01}^T{S_{01}}$ one can get matrix ${L^{( {1,0} )}}$. In general$,$ matrix ${S_{n - 1, n}}$ is the boundary matrix ${B_n}$ between adjacent cliques [4]. If clique $[ {{i_0},\ {i_1}, \ldots ,\ {i_n}} ]$ has a boundary $[ {{i_0},\ {i_1},\ldots ,{i_{p - 1}},\ {i_{p + 1}},\ \ldots ,\ {i_n}} ]$, where ${i_p}$ is removed, then each element of ${B_n}$ is given a plus or a minus sign [6], determined by ${( { - 1} )^p}$, and the resultant matrix is denoted ${S_{[ {n - 1, n} ]}}$, Consequently, the Hodge–Laplacian matrix is obtained by ${L_{( {n, d} )}} = {S_{[ {n, d} ]}}\ S_{[ {n, d} ]}^T$ or $S_{[ {d, n} ]}^T{S_{[ {d, n} ]}}$ (for $n < d$). Note that the non-diagonal elements of a Hodge–Laplacian matrix are not necessarily negative, although all its eigenvalues are non-negative real numbers and its 0 eigenvalue has multiple possibilities (none, one or several).

To introduce some basic operations into networks, define vector spaces ${C_k}$ over the binary field with $k$-cliques as its basis [4]. The addition of two vectors, $c$ and $d$, are defined using the symmetric difference of the two sets, namely $c + d\ = ( {c \cup d} )\ - ( {c \cap d} )$. Thus, chain and cycle can be generalized to a higher-order setting. Next, define a boundary operator acting on adjacent vector spaces, ${\partial _k}: {C_k} \to {C_{k - 1}}$ and define the kernel space, image space and homological group [4]. Then, all $k$-cycles are categorized into equivalent classes. For each class, select one with the shortest length as its representative.

In the homological group, each linearly independent equivalent class of $k$-cycles is called a $k$-cavity. The number of $k$-cavities is given by the Betti number ${\beta _k} = {m_k} - {r_k} - {r_{k + 1}}$, where ${r_k}$ is the rank of the boundary matrix ${B_k}$, with ${r_0} = \ 0$ by convention [4].

How to find all cliques and cavities of a given network? To search for all cliques, there is the Hash graph method [2] and the common-neighbor method [5], whereas to search for cavities, there is the combinatorial optimization method [5] and the potential eigen-spectral method [6]. It is known that the number of zero eigenvalues of the Hodge–Laplacian matrix $\ {L_{( k )}} = \ {L_{( {k, k} )}} + {L_{( {k + 1,\ k + 1} )}}$ equals the number of $k$-cavities, namely the Betti number ${\beta _k}$, where the corresponding eigenvectors provide basic information about the cavities structure.

Finally, with all cliques and cavities found, one can determine the characteristic number $\chi = {m_0}\ - {m_1} + {m_2} - {m_3} + \cdots $, which satisfies the Euler–Poincaré formula: $\chi = {\beta _0} - {\beta _1} + {\beta _2} - {\beta _3} + \cdots $, where ${\beta _0}$ equals the number of connected subgraphs of the network.

The dynamics on simplicial networks, dynamic variables and interactions between nodes are all generalized to simplexes. We consequently study higher-order indexes for measuring the importance of nodes and simplexes. In addition to the known explosive phase transition and bistable behavior in the SIS model [7], the optimal network structure for higher-order synchronizability is worth exploring. We find that higher-order cavities, which play an important role in brain functions [5], have better synchronizability because ${L^{( {0, k} )}} = \ k{L^{( {0,1} )}}$ with larger eigenvalues.

In future research, by replacing nodes and edges in the scale-free model with simplexes, one may introduce preferentially growing simplicial network models [8]. For networks evolving in discrete space-time, there is a need to properly define curvature (related to quantum gravity) satisfying the Gauss–Bonnet–Chern formula. It is also desirable to extend undirected simplicial networks to directed, weighted and multilayered simplicial networks.

***Conflict of interest statement.*** None declared.
